# The Anatomy, Physiology and Pathology of the Human Teeth, with the Most Approved Methods of Treatment; Including Operations and the Method of Making and Setting Artificial Teeth: With 30 Plates

**Published:** 1844-05

**Authors:** 


### REVIEWS.

Art. VI.—*The Anatomy, Physiology and Pathology of the Human Teeth, with the most approved methods of Treatment; including Operations and the method of making and setting artificial teeth: with* 30 *plates.* By Paul B. Goddard, M. D., M.A.N.S., M.A.P.S., Demonstrator of Anatomy in the University of Pennsylvania, Lecturer on Anatomy, &c., &c. *Aided in the practical part* by Joseph E. Parker, Dentist. Philadelphia: Cary & Hart. 1844. 4to. pp. 227.

It was the purpose of the author of this very well executed volume to render it a pandect of dental knowledge. He has collected into it whatever has been ascertained concerning the structure, physiology, and diseases of the teeth, as well as the remedies for the various ills which assail them, and has embellished his work with a profusion of drawings, by which all has been done that art could accomplish to make the subject intelligible. The design of the author seems to us to have been carried out successfully, and with judgment and good taste. The work strikes us as precisely such an one as must delight the heart of the young dentist, who aspires to the reputation of learning in his profession. Nor is this the only class of readers for which this treatise possesses attractions. In this country, where the distinction between physician and general practitioner, and even between physician and surgeon is not known, but every doctor must be at once physician, surgeon, accoucheur, and dentist, some skill in the management, at least in the extraction, of the teeth, is held to be a necessary professional accomplishment. The first operation the student, commonly, is set about performing, after he enters the shop of his preceptor, is pulling teeth. Without any knowledge of their anatomy, his reliance, of course, is on the strength of his *pullekin* and his right arm. In time, and after inflicting much needless pain, and, perhaps, injuring many jaws, he becomes, by dint of mere practice, a good dentist, so far as drawing teeth is concerned. Now a knowledge of what is contained in this compendious work would save patients much suffering, and the young practitioner many mortifications. With this persuasion, and in view of the general necessity there is for physicians engaging more or less in the practice of dentistry, we have undertaken to present an account of such parts of the volume before us as are interesting to the general practitioner.

In a work professing to give a complete account of the teeth, Dr. Goddard felt it proper to begin at the beginning, and hence has devoted a chapter to the history of the art of dentistry. We shall not follow him through this, nor shall we stop to notice the chapters which treat of the anatomy of the maxillary bones, but proceed directly to the teeth.

These important appendages to the human body belong, it seems, to the *dermoid* or cutaneous, and not to the osseous system, as we were taught by Bell, Fox, and others. They are attached to the jaw by the alveoli, which come and disappear with the teeth. Four distinct structures are distinguishable in the teeth, namely: the *enamel*, the *ivory*, the *crusta petrosa*, and the *dental pulp*, which, exhibiting a marked difference in character, require different modes of treatment in the prevention or management of their maladies.

"The enamel is evidently a secretion or crystalization of earthy substances from a solution." It is thickest at the points of the tooth, where its growth begins, by more points than one, and from which it spreads gradually over the whole surface. Where the two formations meet there is a line formed, and if the union between them is not perfect a fissure is left, which often reaches nearly or quite to the ivory, affording a nidus for acid matters which induce decay in the tooth. The enamel is composed of exceedingly minute hexagonal prisms, which under the microscope are seen to be piled obliquely one upon another, waving with a gentle undulation, and presenting an appearance not unlike "the basaltic columns of the giant's causeway." They are held together by animal cement, which amounts to only one per cent. of the substance of the enamel, nearly the whole of the remainder being phosphate and fluate of lime, and carbonate of lime.

The ivory forms the greater part of the tooth, and is usually spoken of as the dental bone. For many reasons, however, Dr. Goddard does not consider it an osseous tissue. He gives in parallel columns the points of difference between bones and teeth, as laid down by Serres. They are as follows:

| A BONE | A TOOTH |
|---|---|
| Passes through a cartilaginous stage. | Passes through no cartilaginous stage, but is a transudation from the surface of the pulp. |
| Has a periosteum. | Has no periosteum. |
| Is affected by rachitis and the other diseases of the osseous system. | Is not affected by rachitis or the other diseases of the bones. |
| Is destroyed by concentrated nitric acid. | Is *not affected* by concentrated nitric acid. |
| When calcined, leaves a white residue. | When calcined, leaves a bluish residue. |
| Is destroyed in extra-uterine conception. | Remains untouched in extra-uterine conceptions. |
| Is vascular. | Is not vascular. |

Bones, moreover, decay much more quickly after interment than teeth, and the diseases of the two tissues offer the least possible analogy.

When a tooth is broken, the ivory presents a white satiny appearance due to its microscopic structure, which consists of tubuli of the 1-4620th of an inch in diameter. An idea of the very small size of these pipes may be conceived when it is stated, that the diameter of the blood globule averages

1-3000th of an inch. The ivory contains more animal matter than the enamel, and consequently a smaller proportion of calcareous matter. It does not lose in bulk or form by being deprived of its earthy ingredients by maceration in a dilute acid. In these respects it resembles bone more than does the enamel, but it still falls short of bone, in animal matter, in the proportion of 28 to 33. It is secreted by the external surface of the pulp, and being destitute of nerves or a circulation undergoes no change when once formed, unless attacked by a chemical or mechanical agent.

The *cementum*, or *crusta petrosa*, the last in its appearance of all the constituents of the teeth, is also a secretion, being deposited by the periosteal covering of the fang. Covering the fang and neck, its thickest point is on the fang, and its thinest where it joins the enamel. In composition it is intermediate between bone and ivory, containing more animal matter than the latter and less than the former.

Of the pulp the following description is given by our author:

"This, in the perfect tooth, is the remains of a substance which once formed the matrix of the growing tooth, taking the form of its ivory, which is secreted from its surface, and finally diminishing in size, so as just to fill the dental cavity. It is composed of a granular matter, which is invested by an exceedingly delicate membrane or *epithelium*, and held together by a delicate cellular tissue. It is highly vascular, receiving an arterial branch from the foramen in the end of the fang, and transmitting its effete blood by a vein, which escapes by the same aperture. An idea may be formed of the tenuity of these vessels by stating, that besides the artery and vein, this foramen (just large enough to admit a human hair) also gives passage to a nervous filament, from the fifth pair or trifacial, which forms a multitude of loops in the substance of the pulp, and confers upon it an exquisite sensibility. The surface of the pulp keeps the ends of the tubuli of the ivory bathed in a transparent and alkaline halitus or serous fluid, which traverses the tubuli by capillary attraction, and keeps the tooth in a moist and *living* state. This degree of vitality, which is very low, assimilates the condition of the tooth somewhat to that of the crystalline humor of the eye, which seems to have a species of independent vitality of a very low grade, living as it were by the imbibition of the *liquor morgagni*, in which it is bathed. The ivory of the tooth formed by the pulp, depends ever after upon its integrity for existence, except as a foreign substance. Its only connexion with the body is by means of the serum filling its tubuli, supplied by the surface of the pulp, and there is but little doubt, that this fluid having reached the under surface of the enamel, continues to pervade it also by means of the delicate membranous matter between its prisms. The cementum possesses minute vessels which penetrate it from the periosteum.

"If the pulp is destroyed by any means, it decays, and the tubuli absorbing a morbid fluid, gives the tooth a dark blue color. Sometimes, when this occurs, the tooth becomes painful and requires extraction; and it is broken, immediately after the operation the decomposing pulp exhales the very quintessence of fœtor. If a tooth be extracted and broken, immediately after death from drowning or strangulation, the pulp will be found almost black from congestion, and sometimes the vessels will be found to be ruptured, and extravasated blood will be discovered between the pulp and the parietes of the dental cavity. The blood globules too large to enter the tubuli, being broken or dissolved, may stain the ivory for some depth. Such cases have been recorded, and have induced the observers to conclude (very erroneously) that ivory was vascular."

So much for the minute anatomy of the teeth. We pass over nearly all that our author gives on this subject, as foreign from our design in the present article; but we cannot forbear to copy his account of the cause of the shedding of the temporary teeth, a subject which has been not a little discussed by physiologists. It is owing, according to his view, to the death of the tooth, produced by the loss of its arterial supply. He remarks:

"When the permanent tooth impinges upon the end of the fang of its predecessor, it cuts off its supply of arterial blood, thus producing its death. This is followed by an absorption of the end of the fang to a greater or less extent, rendering it loose and easily removed. This absorption does not occur in the tooth, as it is now an extraneous body, but is performed by the surrounding tissues, the action of which is excited by the irritation of the rising permanent tooth. During the existence of the temporary set, each side of the lower jaw possesses three arteries, one to supply the bone, a second to supply the germs of the permanent teeth, and a third for the temporary set. When the temporary teeth are shed, this last artery is entirely obliterated."

But we hasten to notice that part of the volume which treats of the diseases of the teeth.

The enamel is subject to fracture, cracking, denudation, and erosion, the remedies for which are various. For cracking, if the fissure be so large as to admit of foreign substances, our author recommends first drying the tooth, and then keeping it moist for a few seconds with a solution of mastic in alcohol, by which the crack will be filled and the ivory defended for a long time from the action of corrosive fluids. The file is to be applied to remove sharp edges of the fracture, but this is an instrument which is always to be applied with caution, since the exposure of the ivory leads to decay and loss of the tooth. For erosion, if deep, plugging is the remedy; but if superficial, treatment is not always required, and it may be sufficient to direct constant washing of the mouth with a weak alkaline solution, which will neutralize any acid secretion and diminish the rapidity of the decay.

Decay, or caries, is the great disease of the teeth, and the principal seat of it is the ivory. It is not caries such as affects the bone, for not being vascular, the ivory is not subject to the pathological changes through which the osseous tissue passes in the process of decay. The decay of a tooth seems to be due to a chemical agency. If from original imperfection in the enamel, or by fracture or erosion, the ivory be denuded, any acid matter coming in contact with it must lead to a wasting of its body. Our author thus relates the series of changes:

"The acid having reached the surface of the ivory by a fissure or hole, commences to act upon its calcareous portion, after neutralizing the alkaline serum which pervades its tubuli.

It first dissolves the calcareous contents of the tubuli, and then the earthy portion of the ivory in their interspaces. The animal portion of the ivory, thus deprived of its calcareous matter, becomes soft, and usually either brown or black, and is gradually mixed with and carried off by the saliva. There is no doubt, at present, that the process is hastened after once an open cavity is formed, by the presence of either animal or vegetable parasites in the decayed cavity, which is an exceedingly proper nidus for such forms, and it is even supposed by Henle (Encyclopedie Anatomique, par A. J. L. Jourdan) that this may account for the spread of the decay from one tooth to another. This process is sometimes exceedingly insidious, in consequence of the original opening in the enamel maintaining its primitive size, until great destruction cf the ivory has taken place beneath it, when it suddenly breaks in and a cavity is formed, where a few hours before none was suspected. In the case where the decay commences on the surface of the ivory under the enamel, the explanation of the process is slightly different. In this case there is no perceptible fissure or hole in the enamel, but a want of union between its basalt-like columns, a porous condition indicated by a slight and scarcely perceptible opacity. The ivory at the bottom of this porous spot is bathed in an alkaline serum furnished by its pulp, whilst the outside of the tooth is constantly exposed to the contact of either acid or acidifying substances. These are the conditions most favorable to the occurrence of *endosmosis* or transudation, the alkaline matter exuding and the acid intruding simultaneously. The ends of the tubuli thus bathed in acid, take it up by capillary attraction and carry it in the direction of the pulp, dissolving the calcareous contents of the tubuli. The process after it has thus commenced, is the same as that just described. The course of the tubuli and their capillary attraction for fluids, causes the decay to progress in the direction of the pulp, hence its investment is soon exposed, and inflammation occurring, toothache is the result. The decay, if suffered to progress, destroys first the crown and pulp, *sometimes* almost without pain, and then becomes nearly stationary, because the sides and not the ends of the tubuli are presented to its exciting cause; the absorption of the acid fluids is therefore very slow, and the rapidity of the decay diminishes in a corresponding ratio."

It is a curious fact, to which our author alludes, that healthy but dead human teeth, when inserted in the place of others that have been lost, are subject to decay, the process being the same as that just described. The inserted tooth presents the same appearances under decay, and the steps by which it is removed do not differ from what are observed to take place in the original tooth. Dr. Goddard justly regards this fact as an incontrovertible argument against the doctrines of Fox, Bell, and others, who attribute decay to an inflammatory action—an action which, if possible in such a structure as the enamel or the ivory at all, is clearly out of the question in the inserted tooth, which is a foreign body.

But if the ivory possesses no sensibility and is not subject to inflammation, it may be asked, how does it happen that the decaying tooth when cut or pressed causes pain? Our author thus explains it:

"The tubuli are softened and converted into a state resembling cartilage; the calcareous matter in their calibres is dissolved and replaced by a morbid fluid, which, when the tubuli are compressed by the touch of an instrument, is injected forcibly on to the surface of the pulp, and produces the pain spoken of. When the decayed and softened part is entirely cut away, this pain ceases, because the parietes of the healthy tubuli are too rigid to allow this compression to take place; this is commonly attributed to the removal of the *sensitive* and *inflamed* bone."

The only remedy for this disease is *plugging*. The decayed part must be removed, and the cavity filled by a substance which will do no injury itself, and will at the same time protect the ivory from all irritating foreign substances. This is an operation for the professed dentist, and we do not propose to give the details of it, but would simply remark, that the only material for filling teeth employed by good operators, we believe, is gold foil. It is in all respects superior to the various other substances which have been used for the purpose, and ought to supersede every other. The mineral cement lately so much vaunted by quacks is discarded by all expert and scientific dentists.

The progress of decay leads finally to exposure of the investing membrane of the pulp, to *odontitis*, and toothache. The inflammation of the pulp, hemmed in as that is by the unyielding walls, is attended by an intensity of pain which few persons will endure for the three days in which it ordinarily runs its course when left to itself. Filling the cavity with cotton imbued with pure creasote is the best palliative remedy; but in order to afford relief the creasote must be genuine. If it has become brown and lost its fluidity it will no longer relieve toothache. Sometimes it is desirable to retain the tooth when all the soothing remedies have failed; in such cases it has become a pretty general practice to destroy the pulp by *arsenious acid.* But it is a practice which is not to be resorted to without caution. Perhaps there is but slight cause to apprehend fatal or serious consequences from the existence of an idiosyncrasy, but the possibility of such a thing should put us on our guard. A case is reported in a late number of the Journal of Dental Science in which arsenic, thus applied, was charged with having led to the death of a valuable member of our profession. The subject was Dr. Wolcott, of Connecticut, in whom erysipelatous inflammation of the face followed the use of arsenious acid in toothache; but the quantity applied was so small that it is not justly chargeable with the fatal result. Not the eighth of a grain was inserted into the cavity of the tooth, with half the quantity of morphine, more than which is often administered internally in intermittents, lepra, and other affections. The smallest dose on record which led to death in the human subject, was four grains and a half, according to Christison; Dr. Beck, however, on the authority of Hahnemann, alleges that two grains will kill a man, and Renault reports that he killed a dog with one grain; but in his experiment the œsophagus of the animal was closed by a ligature, and vomiting rendered impossible. In no instance have serious effects followed the employment of so small a portion as the eighth or tenth of a grain. The death of Dr. Wolcott was clearly the result of erysipelas of the neck and face, in the production of which there is no evidence that the arsenic had an agency. The quantity recommended by our author is the sixteenth of a grain, which is carefully enclosed in the cavity by wax, and would not do any serious mischief if it got into the stomach. He condemns the conjoined use of morphine, which, he says, so far from diminishing, actually increases the pain, while it impedes the escharotic action of the arsenic. Neither does he use the common arsenic of the shops—the article must be pure. It destroys the vitality of the pulp, and also forms with it a compound incapable of putrefaction. For a few hours some pain follows the application, when it ceases, ordinarily, and in a day or two the tooth may be plugged. He adds:

"When the disease has progressed to the suppurative stage, and it is desirable from any cause to save the tooth, it is best done by the application of a minute portion of pure arsenious acid to the dental cavity, which is then covered by wax or a mixture of wax and mastic, and renewed once a week for three or four times. By pursuing this plan the offensive discharge will cease, and the tooth may be plugged and worn for an indefinite length of time."

The last great remedy of all—the dernier resort of the dentist, is extraction. This, like the amputation of limbs, is an evil which is to be put off as long as possible, but in some cases it is the only remedy. How it is to be done, then, becomes the question. Dr. Goddard declares for the forceps in every situation where they can be applied. He is no friend to the key. He exhorts dentists never to take it up, "except when nothing else will answer their purpose." It is apt to fracture the tooth, especially if the claw be either too large or too small, is sure to bruise the gum, and in many cases breaks off the alveolus, causing little flakes of bone to exfoliate for weeks after the operation. "Sometimes the fracture of the alveolus is much more extensive, causing it to be broken away from four or five neighboring teeth. This is always a serious injury, for in the event of hemorrhage it deprives us of the power of using the best remedy, namely, plugging the socket." The forceps, as variously modified, are applicable "to any tooth or fragment of a tooth," and are free from all of the above objections. They should be as small as possible, and skill should supply the place of force. In order to easy extraction, let the gum-lancet be freely used —"it will facilitate *amazingly* the removal of the tooth." The teeth are held in their sockets by ligamentous fibres, which are especially numerous and strong between the molars; these are to be divided by pushing the lancet boldly around in all directions.

The teeth often suffer from a deposit of tartar around their necks. When first deposited it is in the form of a soft yellowish-white substance which may easily be removed by a brush. It soon becomes slightly fetid, and, we are told, if examined by the microscope exhibits a great number of living animalculæ. After a time it hardens, assuming a consistence greater in density than chalk, but never becomes as hard as the enamel. It is composed of the solid portions of the saliva, mucus, and the debris of microscopic animals—or phosphate of lime and magnesia, mucus, and animal matter. The incrustation of tartar causes a separation between the gum and the neck of the tooth. It is to be prevented by the use of the tooth-brush; and when the deposit forms it is to be removed by proper instruments.

Serious hemorrhage sometimes, though we believe rarely, results from the extraction of a tooth. It may arise from a hemorrhagic tendency, of which the records of our profession furnish numerous examples, where profuse and even fatal bleeding follows the slightest wounds; or it may be caused by the failure of the dental artery to withdraw within its bony canal after it has been ruptured. In most instances, this vessel being slightly elongated before it gives way, by which its adhesion to the bone is broken up, retreats into the cavity, and thereupon the bleeding ceases. Where this does not take place and the flow of blood continues too long, or is excessive, it becomes necessary to use measures to restrain it. A stick of nitrate of silver tapered to a point, may be applied directly to the bleeding vessel, and will seldom fail to arrest the hemorrhage; but if it should it is easy to control it effectually by filling the socket with soft linen, cotton, or as is proposed by some, with plaster-of-paris, introduced in a soft state and left to harden in the cavity.

In the greater number of the subjects embraced in this comprehensive work our readers would take no interest, and we therefore pass over them without notice. The professional dentist would not be content with an analytical review of them, however full, and those for whom this article is written are not interested in understanding the various processes of mechanical dentistry. We again refer the young dentist to Dr. Goddard's beautiful volume for information concerning the anatomy, physiology and pathology of the human teeth, as well as the methods of treating their diseases and deformities, and of making and setting artificial teeth, for the illustration of which he will find numerous plates, which not only embellish, but materially enhance the value of the work. It was designed by the author especially for students of dental surgery; but it is not improbable that its instruction would be highly advantageous to many who are engaged in the practice of dentistry. Y.

